# Selected sociodemographic factors and related differences in patterns of alcohol use among university students in Slovakia

**DOI:** 10.1186/1471-2458-11-849

**Published:** 2011-11-09

**Authors:** Rene Sebena, Olga Orosova, Rafael T Mikolajczyk, Jitse P van Dijk

**Affiliations:** 1 Department of Psychology, Faculty of Arts, PJ Safarik University in Kosice, Kosice, Slovak Republic; 2 Department of Educational Psychology and Health Psychology, Faculty of Arts, PJ Safarik University in Kosice, Kosice, Slovak Republic; 3 Kosice Institute for Society and Health, PJ Safarik University, Kosice, Slovak Republic; 4 Department of Clinical Epidemiology, Bremen Institute for Prevention Research and Social Medicine, Bremen, Germany; 5 Department of Social Medicine, University of Groningen, University Medical Center Groningen, Groningen, The Netherlands; 6 Bremen Institute for Prevention Research and Social Medicine, Achterstr. 30, D-28359 Bremen, Germany

## Abstract

**Background:**

Alcohol use and misuse and their relation to sociodemograhic factors are well studied among university students in Western European countries and the USA, but less is known about students in Eastern Europe. The historical past as communistic countries might have affected the social life among these populations, which is again one of the main factors determining the alcohol consumption among university students. The aim of our study was to assess the association of selected sociodemographic factors with different patterns of alcohol use among university students in Slovakia.

**Methods:**

A sample of 813 young adults (mean age 21.1 years, 63.8% females; response rate of 71%) from four universities in Kosice answered questions about their sociodemographic background and about alcohol use. To obtain a detailed picture of different aspects, alcohol use was measured by four variables: frequency of alcohol use, heavy episodic drinking, frequency of drunkenness and problem drinking. Four separate logistic regression models were used to assess the association between sociodemographic and alcohol-related variables. To assess the potentially different effects in both genders, all two-way interactions with gender were tested.

**Results:**

While 41% of the students drank alcohol once a week or more often, 77% reported heavy episodic drinking and 49% had been drunk more than once in the last month. Problem drinking existed in 23.3% of the sample. Gender was consistently associated with all four alcohol-related variables, with males being at higher risk. A higher study year was associated only with lower levels of heavy episodic drinking, but displayed no association with the other studied variables. Living with parents during the semester was consistently associated with less frequent heavy episodic drinking, drunkenness episodes, and problem drinking while having an intimate relationship was associated with less problem drinking only.

**Conclusions:**

Our findings for the university students from Slovakia are in line with previous studies in Western Europe. Additionally, it appears that frequent alcohol use, excessive alcohol use (heavy episodic drinking and drunkenness) and problem drinking among university students represent a continuum and are influenced by the same sociodemographic factors.

## Background

Young adulthood is in many cultures the stage of life in which the highest levels of alcohol consumption occur [1-3]. After entering university, a student's life situation changes, and he or she experiences increased independence, decreased parental guidance, supervision and support, and more social contacts with peers on the university campus. All of these factors potentially contribute to increased alcohol use [4]. Especially important is the fact that alcohol consumption is most often a social activity with peers and therefore forms a cultural event in the process of identity development [5,6]. There are indications that heavy alcohol use at this age is predictive of a range of psychological and physical problems [7]. Alcohol abuse itself, however, is the result of the interaction between personal, environmental and sociodemographic factors [8], a selection of which is presented in the following text. Although there are a number of other risk and protective factors associated with alcohol consumption in young adults, the scope of our analysis covers a subset of sociodemographic variables which are linked to, and seem to be specifically important for, the university environment.

### Gender

A substantial body of research indicates that males are more likely to drink alcohol, consume higher amounts of alcohol and are more likely to be alcohol dependent in comparison with females. These findings are consistent across different countries and cultures [9-11]. Furthermore, the gender difference can also be seen with regard to drinking versus abstinence [12,13], heavy drinking and intoxication [14-16] and alcohol use disorders [17]. Despite the apparent universality of gender differences in drinking behavior, the extent of gender differences may vary across different societies and with regard to different aspects of drinking [16,18]. Gender gaps in the prevalence of heavy episodic drinking for example have become smaller or disappeared in some European countries (such as Ireland, Norway and the United Kingdom) among late adolescents or university students in recent years [19,20].

### Study year

Several studies have shown that students increase their alcohol consumption during their first year at college [21-25]. The first year of studying at a university is an important development period in which students establish identity and social networks, and alcohol use is often part of this process [26]. Being part of a group, or "fitting in," is a major motivating factor for heavy episodic drinking among university students; therefore, it is not surprising that first-year students often socialize in a drinking context and that they make up the largest percentage of party-goers. According to other studies, first-year university students are especially vulnerable to alcohol-caused injuries or death [27,28].

### Parental socioeconomic status

The relationship between parental socioeconomic status (SES) and adolescent alcohol drinking is poorly understood, with inconsistent or even contradictory evidence. While some studies have identified a higher risk of excessive drinking among adolescents from lower SES groups [29,30], other studies have shown an inconsistent relationship between alcohol intake and parental social position [31,32], and still others have found no or even inverse SES gradients in adolescent alcohol consumption [33,34].

### Accommodation during study semesters

University and campus environments include friendship networks and unions in which drinking alcohol is common, endorsed and part of social life. Heavy drinking among students is associated with living away from the parental home in several studies [35-37]. Living in student dormitories, on campuses or in private homes, either with roommates or alone, entails diminishes exposure to parental control and more frequent exposure to peer influences and therefore to opportunities to engage in such problem behaviors as drinking [4].

### Intimate relationship

There is a strong increase in alcohol use during adolescent years [38], but according to Engels and Knibbe, having an intimate relationship does not play a significant role for alcohol consumption during this period [39]. Other studies indicate that relationship formation is associated with lower levels of alcohol use, whereas disruption of the relationship is associated with higher levels of alcohol use [3,40]. Some further studies suggest that couples consisting of two partners who exhibit discordant behavior with respect to heavy-drinking tend to have worse relationships than couples in which only one partner is a heavy drinker [41,42].

### Main goals of this study

Most of the research on drinking behavior among university students was conducted in the USA or in Western Europe [43-45] and comparatively less is known about drinking among students in former communistic countries of Central and Eastern Europe [46]. Some studies show differences across European countries in both, the frequency of alcohol consumption and the proportions of students with problem drinking [1,46]. Based on the past historical experience, there still are differences regarding the social life between countries of Eastern and Western Europe [47]. These differences might also affect social networks among students and consistently reflect on drinking patterns. Patterns of drinking in Central/Eastern Europe are combined with a rather high per capita alcohol consumption. Hazardous drinking patterns are more prevalent in these countries in comparison to Western countries [48], but the knowledge about drinking among university students in the former is still scarce. In order to close this gap in the knowledge, we studied alcohol-drinking behavior among university students in Slovakia. Previous studies on alcohol consumption among students have focused mainly on binge drinking defined as the consumption of at least 4 (females) or 5 (males) consecutive alcoholic drinks per drinking session [49]. Since others [50,51] proposed to use the term "binge drinking" only to describe an extended bout of drinking in which the person neglects other activities in order to drink, we used the term "heavy episodic drinking" to denote high consumption in one drinking session. However, the reported number of drinks per occasion may fail to accurately capture the extent of heavy drinking or drunkenness episodes on college campuses. More importantly, the criterion of heavy episodic drinking may not identify students with a dependency and problem drinking [52,53]. Therefore, this study used several variables to assess the drinking of alcohol among university students: frequency of alcohol use, heavy episodic drinking, frequency of drunkenness, and problem drinking.

The study addresses the following questions: (1) what is the prevalence of specific drinking patterns (high frequency of alcohol consumption, heavy episodic drinking, drunkenness, and problem drinking), (2) are these drinking patterns associated with selected sociodemographic variables (gender, study year, economic status, type of accommodation, and having an intimate relationship) and (3) do the associations between sociodemographic variables and drinking patterns differ for both genders in university students in Slovakia.

## Methods

### Sample and procedure

In 2007 as part of the Cross-National Student Health Study (CNSHS) [54], data were collected in 2007 at three universities in Kosice, Slovakia: the University of PJ Safarik, the University of Veterinary Medicine, and the Technical University. The sample was composed to allow international comparability in CNSHS and was planned to include at least 30% first-year students, about 25% from the social sciences, 25% from the natural sciences, 25% from the law and economy faculties, and 25% from the technical sciences. Under the guidance of field workers a self-administered questionnaire was distributed during regular classes of randomly selected courses for 1st- to 4th-year students. 1140 students were expected in the courses, but only 934 were present. 934 questionnaires were returned and considered for analysis. The proportion of females/males in our sample reflected the proportion of all students in the universities at that time. Among the studied variables, 0.9% responses were on average missing and after listwise deletion was performed for handling missing data, the final sample size was 813 students (response rate 71%). The mean age of the participants was 21.1, SD = 1.8; 63.8% of the respondents were females. All of the asked students completed the questionnaire during a regular 45-minute class period. To increase the accuracy of self-reports, students were assured that their answers would remain confidential. Identification codes and envelopes were also used to emphasize the confidential nature of the survey.

### Informed consent and ethical permission

Participation in the study was voluntary and anonymous. Students were informed that by completing the questionnaire they were providing their informed consent to participate. They were also told that they could terminate the participation at any point while filling out the questionnaire. The permission to conduct the study was granted by the participating institutions: Faculty of Medicine, Faculty of Science, Faculty of Law, Faculty of Public Administration and Faculty of Arts all from the University of PJ Safarik, the University of Veterinary Medicine and Pharmacy in Kosice and the Technical University of Kosice.

### Measures

#### Frequency of alcohol consumption

The frequency of alcohol consumption was measured using the following question: "Over the past three months how often have you drunk alcohol, for example, beer?" The possible answers were: "never," "once a week or less," "once a week," "a few times each week," "every day," "a few times each day". We dichotomized the variable into "drinking less than once a week" versus "drinking once a week or more". The results regarding drinking patterns are reported in Table 1.

**Table 1:** Frequencies of individual levels of drinking patterns prior to dichotomization by gender (*N *= 813)

	Males (%)	Females (%)	Total (%)
**Frequency of alcohol consumption**			

Never	10.2	20.3	16.6

Once a week or less	29.3	49.7	42.3

Once a week	26.0	20.3	22.3

A few times each week	28.7	9.2	16.3

Every day	5.1	0.2	1.9

A few times each day	0.6	0.3	0.5

**Heavy episodic drinking**			

Never	23.0	47.9	38.8

Once	16.1	17.6	17.0

Twice	16.4	16.1	16.3

3-5 times	22.4	14.0	17.0

6-9 times	11.9	3.4	6.5

10 or more	10.1	1.0	4.4

**Alcohol drunkenness**			

Never	34.8	59.8	50.8

1-2 times	42.3	32.8	36.5

3-4 times	14.0	5.7	8.7

5 or more times	8.9	1.0	3.9

**Problem drinking**			

0 positive responses	44.1	67.6	58.8

1 positive response	23.6	18.2	20.1

2 positive responses	18.4	8.7	12.5

3 positive responses	9.1	4.6	6.2

4 positive responses	4.8	0.9	2.3

#### Heavy episodic drinking

The frequency of heavy episodic drinking was measured by asking: "Think back again over the last 30 days. How many times (if any) have you had five or more drinks on one occasion?" (A "drink" is a glass/bottle/can of beer (ca 50 cl), a glass/bottle/can of cider (ca 50 cl), 2 glasses/bottles of alcopops (ca 50 cl), a glass of wine (ca 15 cl), a glass of spirits (ca 5 cl) or a mixed drink). The options for answers were "never," "once," "twice," "3-5 times" "6-9 times" and "10 or more times." We classified respondents into non-episodic drinkers (if they responded "never") and heavy episodic drinkers (all others).

#### Alcohol drunkenness

To identify students with higher risk behavior who drink to excess or to get drunk, we used the question "How many times have you been drunk during the last four weeks?" The options for answers here were: "never", "once or twice", "3-4 times," and "5 or more times." Responses were dichotomized into "never" versus all other.

#### Problem drinking

Finally, to gather data on problem drinking we included an alcoholism-screening test, the CAGE test [55]. CAGE is a brief screening instrument consisting of four questions (Have you ever felt you should **C**ut down on your drinking? Have people **A**nnoyed you by criticizing your drinking? Have you ever-felt bad or **G**uilty about your drinking? Have you ever had a drink in the morning to get rid of a hangover? (**E**ye opener). Each question is answered either "yes" or "no." Two or three affirmative answers suggest problem drinking, while four positive responses raise a serious suspicion of alcohol dependence. Mean inter-item correlation (MIIC) was 0.26 (according to the guideline of Briggs & Cheek [56] the MIIC should range above 0.20). We classified the respondents as non-problem drinkers (less than two positive answers) and problem drinkers (two or more positive answers).

#### Sociodemographic variables

Gender and study year were based on individuals' self-reports on the questionnaire. A respondent's socio-economic status (SES) was assessed using two measures: parental education and the self-perceived income sufficiency of the student. We first asked about the father's and mother's educational status separately--"What is the highest education level of your mother, father?"--with these answer options: "No formal education," "Secondary vocational school," "A levels," "Bachelor's degree," "Master's degree and Ph.D. or equivalent." The educational levels of parents were subsequently collapsed, for the purpose of analysis, into two categories: low (A levels and lower degree) versus high (bachelor's and higher degree). Afterwards, the educational levels of parents (low vs. high) were combined into the following four groups: both parents high, both parents low, mother high and father low, and father high and mother low. Perceived income sufficiency was measured by asking: "How sufficient do you consider your income?" with four Likert scale responses ("always sufficient," "mostly sufficient," "mostly insufficient" or "insufficient") which were dichotomized into "always sufficient" versus "other." Students were also asked about the type of accommodation they lived in during the semester. The responses were dichotomised into "I live with my parents" versus "I do not live with my parents." Finally, participants were asked whether they were currently in an intimate relationship.

### Statistical analysis

First, we conducted a descriptive analysis of the study population. Next, we used the phi coefficient to assess the correlation between the dichotomised variables related to alcohol [57]. Phi values from -1.0 to -0.7 indicate a strong negative association, -0.7 to -0.3 a weak negative association, -0.3 to +0.3 little or no association, +0.3 to +0.7 a weak positive association, and +0.7 to +1.0 a strong positive association. The independent association between five variables (gender, study year, parental educational status, having an intimate partnership, type of accommodation) and alcohol-related variables (frequency of alcohol consumption, heavy drinking, drunkenness, problem drinking) was studied in four separate logistic regression models. To assess the potentially different effects in both genders, all two-way interactions with gender were tested in each model separately. The results were reported as odds ratios (OR) with 95% confidence intervals (CI). The analysis was performed using SPSS 16.

## Results

### Description of the sample

The composition of this sample was as follows: 43.3% of students came from the University of PJ Safarik; 16.5% from the University of Veterinary Medicine and 40.2% from the Technical University. There was a gender distribution of 63.8% females and 36.2% males. Other main descriptive characteristics of the study population are presented in Table 2.

**Table 2:** Characteristics of the study sample and alcohol-related variables across these characteristics (*N *= 813)

	Total		Alcohol consumption		Heavy episodic drinking		Drunkenness episodes		Problem drinking (CAGE)	
			**Reporting high frequency**		**Reporting high frequency**		**Reporting high frequency**		**Problem drinkers**	

	**N**	**%**	**N**	**%**	**N**	**%**	**N**	**%**	**N**	**%**

**Gender**										

Female	519	63.8	156	30	270	52.1	207	39.8	74	14.3

Male	294	36.2	178	60.5	226	77	192	65.2	95	32.3

**Study year**										

1st year	278	34.2	107	38.5	175	62.9	140	50.3	59	21.2

2nd year	221	27.2	109	49.6	144	65.3	114	51.8	48	21.9

3rd year	93	11.4	38	40.6	52	55.9	39	42.2	26	28.4

4th year	221	27.2	80	36.2	94	42.7	105	47.6	36	16.5

**Parental educational status**										

Both parents low	445	54.8	175	39.4	260	58.5	206	46.2	93	20.9

Mother high, father low	70	8.6	32	45.0	47	66.7	33	47.4	10	14.3

Father high, mother low	120	14.7	48	39.6	73	60.9	58	48.5	20	16.3

Both parents high	178	21.9	80	45.0	117	66.0	100	56.0	48	26.7

**Perceived income sufficiency**										

Always sufficient	550	67.7	228	41.5	342	62.1	272	49.5	117	21.2

Other	263	32.3	123	46.6	163	62.1	135	51.3	68	25.8

**Accomodation during semester**										

With parents	393	48.4	154	39.2	224	56.9	186	47.3	74	18.8

Other	420	51.6	178	42.4	274	65.2	210	49.9	97	23.1

**Intimate relationship**										

Yes	454	55.8	165	36.3	273	60.2	211	46.5	78	17.1

No	359	44.2	177	47.9	224	62.3	189	52.7	96	26.7

A total of 41.1% (60.5% males vs. 30.0% females) of the sample drank alcohol once a week or more often. 77.0% of males and 52.1% of females were heavy episodic drinkers (consuming 5 or more drinks on a single occasion), while 49.1% (65.2% males vs. 39.8% females) had been drunk more than once during the last month. Problem drinking (two or more positive responses in CAGE) was estimated for 32.3% of males and 14.3% of females.

After correlating all drinking-related variables, we found that the strongest relation was between heavy episodic drinking and frequency of drunkenness (phi = 0.62 for males and phi = 0.64 for females). Correlations between problem drinking and other alcohol-use-related variables were relatively weak, for example for frequency of drinking and problem drinking (phi = 0.19 for males, phi = 0.28 for females) and heavy episodic drinking and problem drinking (phi = 0.2 for males and phi = 0.21 for females). These findings justify the analyses of the alcohol-related variables in separate models.

### Variables associated with alcohol-use-related variables

The results from multivariable logistic regression models are summarized in Table 3. The analyses revealed that gender was consistently associated with all four alcohol-use-related variables. Female students were less likely to report frequent consumption of alcohol, heavy episodic drinking, drunkenness episodes or problem drinking than male students. On the contrary, some other variables were only associated with a single alcohol-related variable. The study year was associated only with heavy episodic drinking, which was less frequent in higher study years. Students of parents with the same educational level, either high or low, were more likely to be involved in problem drinking than those from families in which one parent had a higher and one a lower education (regardless of whether the mother or the father had the higher education). The perceived income was not associated with any of the alcohol-related variables studied in this analysis. Living with parents during the semester was associated with less frequent heavy episodic drinking, drunkenness and problem drinking, but not with the frequency of drinking. The odds of problem drinking were lower for those with an intimate partner than for singles.

**Table 3:** Factors independently associated with alcohol use variables in university students

	High drinking frequency	Heavy episodic drinking	Drunkenness episodes	Problem drinking (CAGE)
	OR (95%CI)*	OR (95%CI)*	OR (95%CI)*	OR (95%CI)*

**Gender**				

Females	**0.28 (0.20-0.39)**	**0.26 (0.18-0.37)**	**0.34 (0.25-0.48)**	**0.39 (0.27-0.57)**

Males	1	1	1	1

**Study year**	0.96 (0.84-1.11)	**0.87 (0.77-0.98)**	0.94 (0.83-1.07)	0.97 (0.83-1.14)

**Parental educational status**				

Both parents high	1.41 (0.94-2.11)	1.42 (0.93-2.15)	1.34 (0.90-1.99)	**1.70 (1.09-2.64)**

Mother high, father low	1.17 (0.68-2.00)	1.38 (0.79-2.40)	1.02 (0.61-1.72)	0.64 (0.32-1.30)

Father high, mother low	0.88 (0.56-1.40)	1.01 (0.69-1.73)	0.96 (0.62-1.49)	0.69 (0.39-1.23)

Both parents low	1	1	1	**1**

**Perceived income sufficiency**				

Always sufficient	0.82 (0.58-1.15)	1.01 (0.72-1.43)	0.90 (0.65-1.26)	0.73 (0.49-1.08)

Other	1	1	1	1

**Accomodation during semester**				

With parents	0.74 (0.54-1.02)	**0.61 (0.44-0.85)**	**0.73 (0.54-0.99)**	**0.68 (0.48-0.97)**

Other	1	1	1	1

**Intimate relationship**				

Yes	0.75 (0.54-1.04)	1.06 (0.76-1.50)	0.83 (0.60-1.15)	**0.64 (0.45-0.92)**

No	1	1	1	1

**C-statistics**	0.70	0.68	0.69	0.68

**Nagelkerke's R-square**	0.149	0.131	0.101	0.122

* adjusted for all variables in the table

There was no significant interaction between gender and the independent variables in any of the models.

To assess the potentially different effects in both genders, interactions of all other sociodemographic variables with gender were tested, which revealed no significant (*p *< 0.05) interactions with respect to any alcohol-related variable.

## Discussion

To gather data on alcohol consumption and problem drinking among university students in Slovakia we used four alcohol-related variables, which measure different aspects of drinking. Frequency of drinking, which is the most general indicator, does not assess the quantity of consumed alcohol. As it is such a broad measurement, a high reported frequency presents only a relatively small concern. However, it was demonstrated that alcohol-related health and social problems tend to increase as the frequency of alcohol consumption rises [58]. Heavy episodic drinking and the frequency of episodes of drunkenness are both measures which provide useful information for detecting more hazardous drinking.

We found that 60.5% of all males drank once a week or more often, and that 77.0% of males reported heavy episodic drinking. A similar pattern was found for female students. This means that some students drink infrequently, but if they do, they drink a lot. Also other studies found heavy episodic drinking to be a very common pattern among university students [59]. In general, our findings indicate a high frequency of drinking, heavy episodic drinking and drunkenness, as well as problem drinking among university students.

### Gender

Among the studied factors, gender had the strongest association with all alcohol-related variables, with males being at higher risk, which is in contrast to some studies indicating a declining difference between genders in alcohol-related variables [60,61]. However, our results are consistent with observations from many previous studies [62-65]. In the literature, the most common explanation for why males and females differ in their drinking behavior is that alcohol consumption symbolizes and enhances male's greater power in relation to females [66,67]. From a biological point of view, females have lower rates of gastric metabolism of alcohol than males [68,69] and smaller volumes of body water in which the alcohol is distributed [70,71]. Thus females may need to consume less alcohol than males to derive the same effects and may be more likely than males to experience unpleasant acute effects from alcohol [72]. Apparently, these patterns and explanations are still valid in the Slovak student population, in contrast to findings from some Western European countries [39]. On the other hand, we found no interactions between gender and the other sociodemographic variables considered in this analysis, indicating that the effects of other variables on drinking do not differ strongly by gender.

### Study year

Based on the assumption that the overall drinking behavior of university students has not changed in recent years, we found only partial evidence of a gradual change in alcohol drinking during the four university years. In our study, the academic year was associated only with heavy episodic drinking. The finding that students from higher study years are less involved in heavy episodic drinking than the students from lower study years may indicate that either the pattern of drinking turns out to be more stable as the students get used to the cultural norms of university life as time passes or that heavy episodic drinking becomes more prevalent in the new generation of university students. For all other alcohol-related variables, we did not find any significant differences across the university years. This is consistent with other findings related to the development of students during university years [73,74].

### Socioeconomic status

Evidence on the relationship between SES and health risk behaviors in adolescence is often inconsistent or even contradictory. This study investigated two different dimensions of SES separately: parental education and students' perceived income sufficiency. According to our results, only students from both extreme groups--highly-educated families (both parents highly educated) as well as lowly-educated families (both parents with a low education level)--faced a higher risk of problem drinking. If students from higher SES families experienced more restraints during adolescence, they might be more prone to excessive drinking when gaining independence. On the other hand, students from lower SES groups might experience a more permissive environment with regard to alcohol and develop problem drinking [75]. In families with differing levels of parental education, there was no difference, regardless of which of the parents achieved the higher level of education. We additionally assessed the effects of perceived income sufficiency on drinking behaviors and observed no association. An explanation for this could be that alcohol is relatively cheap and is easy to access, and that drinking on university campuses is a social activity and students having less money may still be invited by others to go out drinking.

### Living at the parental home

Leaving the parental home often coincides with an increase in heavy alcohol use [65]. We found that accommodation is an important risk factor for heavy episodic drinking, alcohol drunkenness and problem drinking among university students. Also, other authors have found an association between the social environment of university life and student drinking [62,76,77]. Probably, the reason for this is a strong response to the social environment (socialization effect). The proximity to parents appears to play a role in protecting students from alcohol problems, as evidenced by the lower rates of drinking problems among students who live with their parents. Parents probably do not tolerate negative alcohol-related behaviors, and they are also able to monitor students who live at home more than those who do not live at home [78]. We did not find differences in the frequency of drinking between students living in the parental home and those who did not. However, as stated above, this is the least strong indicator of alcohol-related risk behavior, and the lack of a difference might be caused by social drinking at the parental home.

### Intimate relationship

We found that having an intimate partner was associated with two alcohol-related variables: respondents with an intimate partner were less involved in frequent drinking and problem drinking than students who were not in an intimate relationship. Although the findings for other drinking patterns were not significant, the same trend was observed for frequency of drunkenness. As a potential explanation of this phenomenon, Silbereisen suggested that involvement in a relationship is accompanied by changes in leisure activities; partners go to pubs or discos less often and seek each other's company in private settings [79]. Another explanation may be that one would not tolerate his/her partner's heavy drinking and students with frequent episodes of heavy drinking are less likely to have stable partnerships.

### Limitations

In this survey sociodemographic correlates of four different patterns of alcohol use among university students in Slovakia were studied. Given the self-reported measures of drinking, some underreporting, for example for problem drinking, which is socially undesirable, might have occurred. In line with the National Survey on Drug Use and Health we used the same criterion (5 or more drinks) for measuring heavy episodic drinking for both genders, while many authors argue that four drinks or more should be used for females [26,80]. Due to different physiology, females reach higher blood alcohol concentration levels compared to males after consuming equivalent doses of alcohol and this might have resulted in an underestimation of heavy episodic drinking in female students.

A further limitation is the cross-sectional design, which makes impossible to formulate conclusive statements about causality. We cannot exclude possible biases regarding missing or incorrect information due to social expectation bias in self-reported data, but we made several steps to guarantee confidentiality, which typically reduces social expectation bias. Since the response rate was relatively high and the survey covered different areas and not only the here studied questions, selection bias is likely limited. Some measures used in our study are short and might not have derived the whole information. For example, socio-economic status was measured only indirectly. There are also limitations related to the representativeness of the sample of the present study for all students in Slovakia. While we studied a relatively systematic sample from three universities in one town, the prevalence of alcohol use may be different in other parts of Slovakia.

## Conclusion

Overall, the current study confirmed associations between alcohol use and problem drinking and some sociodemographic factors in university students in Slovakia which is in agreement with studies from other populations. It seems that a different historical past did not influence patterns of alcohol use, however, we cannot say whether the situation observed in 2007 is a consequence of recent adaptations or was already in place before the political changes of the early 1990s. Additionally, our findings indicate that frequent alcohol use, excessive alcohol use (heavy episodic drinking and drunkenness), and problem drinking (CAGE) among university students represent a continuum and are influenced by the same sociodemographic and psychosocial factors. Male gender and living in a university campus environment were associated with excessive and problem alcohol use patterns. Another factor that affects student problem drinking is an intimate relationship. For the study year and the SES we were not able to confirm the expected associations, however. These findings should be taken into account when developing prevention programs.

## Competing interests

The authors declare that they have no competing interests.

## Authors' contributions

RS developed the research question, conducted the analyses and drafted the manuscript. OO and JPvD commented extensively on the manuscript. RTM developed the study design, supervised the analysis, contributed to interpretation and writing. All authors read and approved the final manuscript.

## Pre-publication history

The pre-publication history for this paper can be accessed here:

http://www.biomedcentral.com/1471-2458/11/849/prepub
